# In Vivo Photoacoustic Ultrasound (PAUS) Assay for Monitoring Tendon Collagen Compositional Changes during Injury and Healing

**DOI:** 10.3390/diagnostics14141498

**Published:** 2024-07-12

**Authors:** Joseph B. Newton, Courtney A. Nuss, Stephanie N. Weiss, Rebecca L. Betts, Chandra M. Sehgal, Louis J. Soslowsky

**Affiliations:** 1 McKay Orthopaedic Research Laboratory, Department of Orthopaedic Surgery, University of Pennsylvania, Philadelphia, PA 19104, USA; josephbnewt@gmail.com (J.B.N.); cnuss@pennmedicine.upenn.edu (C.A.N.); westep@pennmedicine.upenn.edu (S.N.W.); rebecca.betts@pennmedicine.upenn.edu (R.L.B.); 2 Department of Radiology, University of Pennsylvania, Philadelphia, PA 19104, USA; chandra.sehgal@pennmedicine.upenn.edu

**Keywords:** photoacoustic imaging, Achilles, in vivo, rat, endogenous, collagen

## Abstract

Tendon injury and healing involve significant changes to tissue biology and composition. Current techniques often require animal sacrifice or tissue destruction, limiting assessment of dynamic changes in tendons, including treatment response, disease development, rupture risk, and healing progression. Changes in tendon composition, such as altered collagen content, can significantly impact tendon mechanics and function. Analyses of compositional changes typically require ex vivo techniques with animal sacrifice or destruction of the tissue. In vivo evaluation of tendons is critical for longitudinal assessment. We hypothesize that photoacoustic ultrasound detects differences in collagen concentration throughout healing. We utilized photoacoustic ultrasound, a hybrid imaging modality that combines ultrasound and laser-induced photoacoustic signals to create detailed and high-resolution images of tendons, to identify its endogenous collagen composition. We correlated the photoacoustic signal to picrosirius red staining. The results show that the photoacoustic ultrasound-estimated collagen content in tendons correlates well with picrosirius red staining. This study demonstrates that photoacoustic ultrasound can assess injury-induced compositional changes within tendons and is the first study to image these targets in rat Achilles tendon in vivo.

## 1. Introduction

Tendon is an important mechanical tissue, crucial for musculoskeletal function, and is known for its strong structure–function relationship. The extracellular matrix (ECM) is primarily made up of a collagen 1 network in an aligned hierarchical structure and is organized into bundles called fibrils. This structure promotes even force distribution and contributes to the tendon strength and stiffness [1]. Tendon disorders make up over 30% of musculoskeletal consultations, ranging from debilitating ruptures to chronic degeneration, often leading to significant healthcare costs and reduced quality of life [2]. Despite surgical interventions, the recovery process is often prolonged and incomplete, with a high retear rate and a failure to fully restore the tendon’s original structure and function [1,3]. After a prolonged healing process, tendons never fully recapitulate their native structure and function [4]. Studies have shown a between 20 and 80% decrease in mg/g dry weight of total collagen lasting through the inflammatory phase of tendon healing, which can be maintained well into the proliferative phase [5,6,7]. At later stages, total collagen dry weight never reaches native tendon levels, plateauing at 90% of original levels [8].

In vivo assessment of tendons is critical for evaluating changes in the biological environment, composition, and function during healing. Longitudinal, in vivo analysis of tendons can aid in evaluating the response to treatment, development of disease, risk of rupture, and progression of healing [9]. Conventional in vivo analyses of tendons include functional measures such as patient questionnaires, tendon strength, and range of motion, structural assessment using MRI or ultrasound, and vascularity with various ultrasound modalities such as color Doppler [10]. However, these methodologies cannot quantify many compositional changes that occur in tendons during healing, tendinopathy, and disease [11].

Endogenous photoacoustic ultrasound is characterized by imaging a tissue absorber already present within the tissue of interest without the use of contrast agents. Common endogenous targets include hemoglobin, collagen, melanin, lipids, calcium, and water, each with a unique absorption spectrum [12]. Oxygenated and deoxygenated hemoglobin have been measured in tendons in vivo, targeting measures for tendon vascularity throughout healing [13,14]. Although hemoglobin has signal strength within the near-infrared region (NIR) of 680–980 nm, collagen has been measured at 725 nm, 808 nm, 875 nm, 920 nm, and 980 nm in multiple tissue types [15,16,17,18,19].

The objective of this study was to assess the novel application of endogenous photoacoustic ultrasound in tendons using an acute Achilles rupture as a model for disruption to the tissue composition. Specifically, this study evaluated the accuracy and sensitivity of measuring endogenous collagen. We hypothesized that detectable differences in collagen concentration could be measured using photoacoustic ultrasound throughout tendon healing.

## 2. Materials and Methods

### 2.1. Study Design

**Figure 1:**
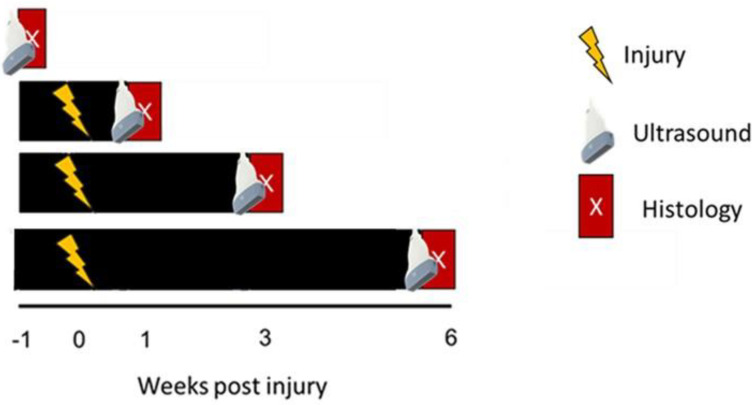
Study design schematic for photoacoustic ultrasound evaluation. The left limbs of eight animals were utilized for photoacoustic ultrasound targeting collagen with histology as a comparison assay. At all time points, animals were sacrificed immediately following in vivo measures for ex vivo analyses.

The accuracy and sensitivity of photoacoustic ultrasound were evaluated using an acute injury of the rat Achilles tendon. Collagen was used to assess the compositional changes in tendons following acute injury. This study utilized 32 Sprague Dawley rats at 6 months of age under IACUC approval (Figure 1). These animals were divided into four groups, one for each key injury time point: (1) pre-injury, (2) 1 week post-injury, (3) 3 weeks post-injury, and (4) 6 weeks post-injury. Each animal underwent bilateral Achilles tendon complete transection followed by immobilization for 1 week. Left limbs (n = 8/time point) were used for photoacoustic ultrasound quantification of collagen content, with comparisons to picrosirius red staining.

### 2.2. Injury Model

Following aseptic technique, rats were anesthetized under general anesthesia (isoflurane), and the left hindlimb was shaved and disinfected. Briefly, a 3 mm longitudinal incision was created from the Achilles myotendinous junction to the osteotendinous junction, thus providing exposure to the Achilles tendon. The tendon was then bluntly transected using the back of a scalpel blade, tearing the fibers approximately 6 mm from the calcaneal insertion [19]. Skin was sutured and the limb was immobilized in plantarflexion for 7 days, followed by a return to cage activity.

Immediately following surgery, rat limbs were immobilized with a Robert Jones bandage with a rigid splint and the injured hind limb was wrapped with gauze and vetwrap. The digits were left exposed for monitoring of limb perfusion. Lastly, a coating of polymethyl methacrylate (PMMA) was applied to the entire cast. Casts were checked daily and replaced as needed. This technique maintains an approximation of severed tendon ends, mimicking the clinical situation better than neurologically induced disuse.

### 2.3. Endogenous Photoacoustic Ultrasound

**Figure 2:**
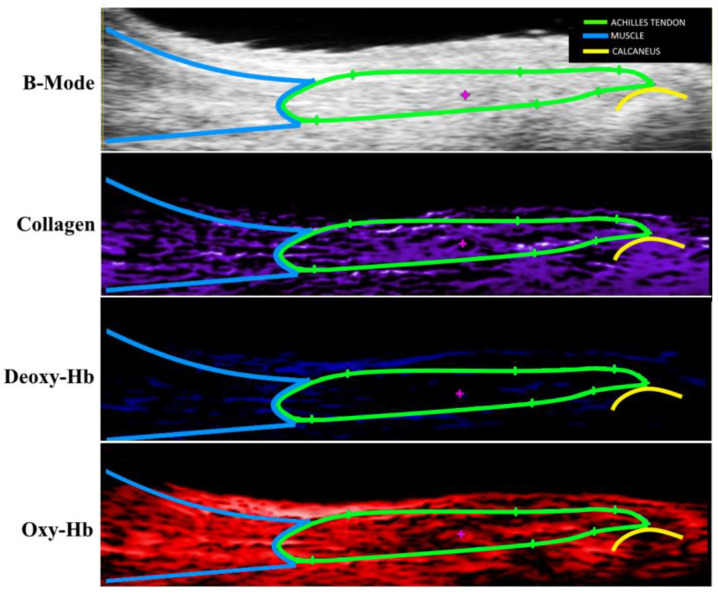
B-mode and photoacoustic ultrasound images of an uninjured Achilles tendon in the sagittal plane. Gastrocnemius is highlighted in blue, the Achilles in green, and the calcaneus in yellow.

Rats were anesthetized with isoflurane under medical air (normoxia). Hair was removed from the hind limb by shaving and hair removal cream. The animal was placed on a heated imaging table. To consistently load the tendon between groups, the ankle was secured at 90° flexion. The transducer was placed to image the Achilles tendon in the sagittal plane in a single section, the transducer remained in a fixed position at the midline during the tendon imaging (Figure 2). Photoacoustic imaging was acquired with the Vevo LAZR Photoacoustics Imaging System (VisualSonics Inc., Toronto, ON, Canada), using a 50 MHz linear array transducer (LZ550) (VisualSonics Inc., Toronto, ON, Canada) with an axial resolution of 44 μm and a wavelength range of 680–970 nm. Images were taken through the entire range of available wavelengths at 5 nm intervals. Time gain composition (TGC) and image acquisition settings were held constant for all specimens (PA gain—52 dB, 2D gain—20 dB, image depth—12 mm, image width—14 dB).

Using spectral unmixing, the contributions of collagen, and oxygenated and deoxygenated hemoglobin to the overall signal were calculated in the VevoLab software (V 5.5.0.295) package for photoacoustic analysis (VisualSonics Inc., Toronto, ON, Canada). The acquired photoacoustic signals ranged from 680 to 970 nm, encompassing the known absorption spectra for each component from the phantoms. The photoacoustic signal at each wavelength was treated as a linear combination of the component spectra, weighted by their relative concentrations. By applying a linear unmixing model, the concentration map of each component was generated, which allowed for differentiation and visualization of the distributions of collagen, and oxygenated and deoxygenated hemoglobin. It is important to note that the selection of each component directly impacts the results of spectral unmixing, as the model will only be able to detect designated components. Signal strength, measured in arbitrary units, and tissue area highlighted for each signal were used to compare between groups. PA Avg, the average photoacoustic signal for each component within the region of interest, was compared to each assay.

### 2.4. Picrosirius Red Histology

**Figure 3:**
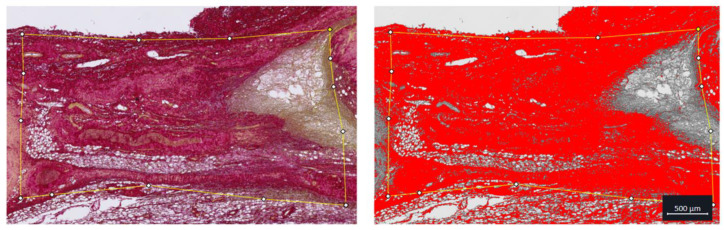
Picrosirius red staining and quantification. Example image of picrosirius red staining (**left**) and positive staining identification (**right**) of the injury site.

Following sacrifice, tendons were excised, and histologic samples were processed. Samples were immersed in 10% neutral-buffered formalin for immediate fixation for 24 h. Samples were then decalcified in Immunocal for 7 days, embedded in paraffin, and sectioned at 10 μm before staining with picrosirius red (PSR) and being imaged under bright field. Picrosirius red staining identifies collagen content within the tissue. Regions of interest were identified for the whole tendon and the injury site. The area of positive stain was calculated in ImageJ as the percentage of positive pixels in the green channel over the total number of pixels for the tendon and presented as percent area (Figure 3).

### 2.5. B-Mode Echogenicity Analysis

B-mode image analysis was performed using a custom MATLAB (Mathworks, Natick, MA, USA) script, using a previously described method [20]. Briefly, collagen fascicles appear hyperechoic under ultrasound, while the non-collagenous matrix appears hypoechoic. More aligned tissue appears hyperechoic, as well. Echogenicity was defined as the grayscale average values for each B-mode image, which were calculated using a custom MATLAB script.

### 2.6. Statistics

Correlation analyses were performed with matched samples for each assay. All measurements were made with regions of interest for the whole tendon and the injury site. PA Avg, the average photoacoustic signal for each component within the region of interest, was compared to each assay. Following correlation analysis, data were separated by each healing time point and analyzed. Data sets were tested for normality using D’Agostino–Pearson, Anderson–Darling, and Shapiro–Wilk normality tests. For normal data, one-way ANOVAs with Bonferroni corrections were conducted for pre-injury, and 1, 3, and 6 weeks post-injury groups. For non-parametric data, Kruskal–Wallis tests were used to compare between time points. Significance was determined by *p* < 0.05 for all analyses. Outlier analysis was performed and determined by +/− >2 standard deviations away from the mean. Unfortunately, one animal in the 1-week group died before data collection; therefore, no data were obtained from this subject and for this group, n = 7.

## 3. Results

### 3.1. Collagen Photoacoustic Signal Exhibited a Strong, Positive Correlation with Picrosirius Red Staining

**Figure 4:**
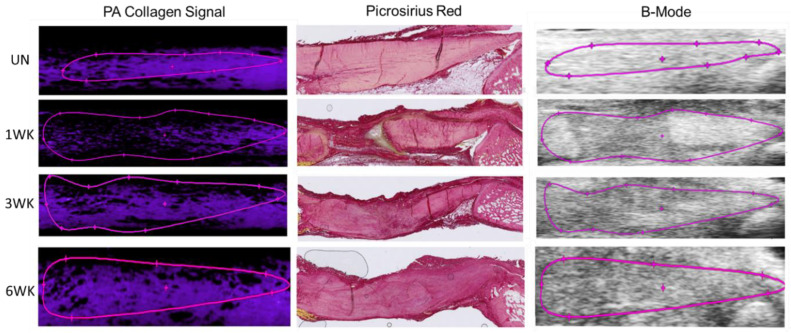
Representative images for each modality used to assess collagen content within the tendon, separated by time point. Purple line denotes tendon ROI.

**Figure 5:**
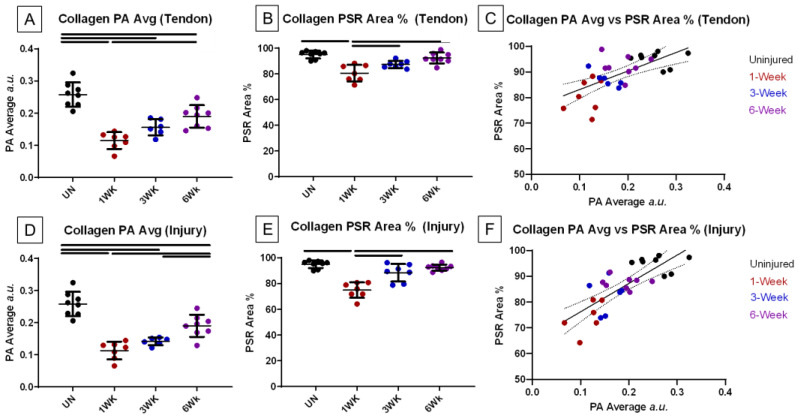
Average photoacoustic signal for collagen, picrosirius positive stain, and their correlations. (**A**,**D**) Measured photoacoustic average signal of collagen for whole tendon and injury site, respectively. (**B**,**E**) Picrosirius red positive stain for whole tendon and injury site. (**C**,**F**) Correlation comparisons of photoacoustic average and picrosirius red positive stain for whole tendon and injury site. (**A**,**B**,**D**,**E**): Data presented as mean ± standard deviation. Solid bars denote significance. Significance set to *p* < 0.05. (**C**,**D**): Solid line denotes line of best fit, dashed lines denote 95% confidence interval.

To assess the ability of endogenous photoacoustic ultrasound to estimate collagen content within the tissue, the photoacoustic average signal, picrosirius red-positive area, and B-mode echogenicity were examined for the whole tendon and the injury site alone (Figure 4). For the whole tendon and injury site alone, the average photoacoustic signal intensity (PA Avg), showed significant decreases between uninjured values and all time points post-injury and a significant increase from 1 week post-injury to 6 weeks post-injury (Figure 5A,D). PA Avg also significantly increased from 3 to 6 weeks post-injury in the injury site alone. Overall, positive picrosirius red staining did not show as many significant changes between groups; however, there were significant differences between 1 week post-injury and all other groups analyzed for both whole tendon and injury site measurements (Figure 5B,E). Correlative statistics were calculated for PA Avg and PSR Area % (Figure 5C,F, Table 1). A significant correlation was observed between the PA Avg measurements and the PSR Area % in both tendon and injury sites. A moderately strong positive correlation coefficient (r) of 0.64 was found between PA average measurements and PSR area percentages in the tendon (*p* < 0.001). Similarly, for injury sites, a stronger positive correlation was observed (r = 0.72, *p* < 0.0001). These results suggest that PA measurements are strongly associated with collagen content in both whole tendon and injury sites.

### 3.2. Some Variance in Photoacoustic Signal vs. PSR Area Is Likely Due to Differences in Collagen Alignment

**Figure 6:**
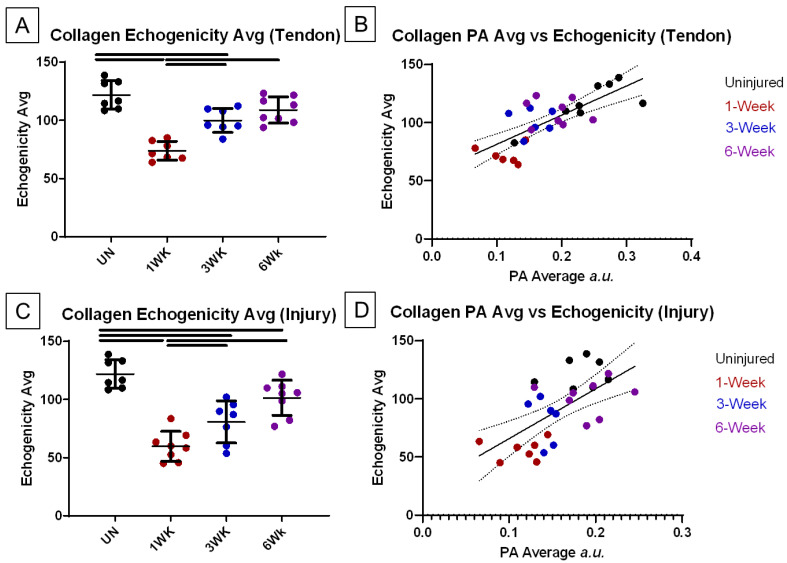
B-mode echogenicity average throughout healing and its correlation to collagen average photoacoustic signal. (**A**,**C**) Average B-mode echogenicity for whole tendon and injury site, respectively. Data presented as mean ± standard deviation. Solid bars denote significance. Significance set to *p* < 0.05. (**B**,**D**) Correlation comparisons of PA Avg and echogenicity avg. for whole tendon and injury site. Solid line denotes line of best fit, dashed lines denote 95% confidence intervals.

To help understand the contribution of collagen alignment to the collagen photoacoustic signal, the average photoacoustic signal and echogenicity were compared for each animal (Figure 6, Table 2). When segmented by time point, echogenicity was significantly decreased from pre-injury values at 1 to 3 weeks post-injury and increased between 1 and 3 weeks post-injury in the whole tendon (Figure 6A). The injury site alone showed similar differences, in addition to a significant decrease from uninjured to 6 weeks post-injury (Figure 6B). Correlative statistics for PA Avg and echogenicity average showed significant correlations for both tendon and injury sites (Figure 6B,D, Table 2). In the case of the tendon, there was a strong positive correlation (r = 0.75, *p* < 0.0001) between PA and echo averages, indicating a high degree of association between these variables. In injury sites, a similar positive correlation was also observed (r = 0.60, *p* = 0.0003), albeit slightly lower.

## 4. Discussion

The objective of this study was to explore a novel application of photoacoustic ultrasound as an in vivo technique for assessing tendon composition. The endogenous photo absorber collagen was targeted. The photoacoustic imaging results were compared with established histological and imaging techniques. These results indicate that collagen content estimated by photoacoustic signal correlates strongly with picrosirius red staining, and the variation between the measurements is likely due to a decrease in overall ultrasound signal from unaligned collagen fibers, as evident by a strong correlation between the photoacoustic signal and echogenicity. This study yielded compelling evidence that photoacoustic ultrasound can be used in vivo to evaluate changes in collagen composition within the tendon non-invasively.

The results demonstrate that collagen content can be estimated over the course of tendon healing in a rat model of acute Achilles rupture using photoacoustic ultrasound. We induced an Achilles rupture, identified key time points to represent each phase of tendon healing, and compared photoacoustic measurements to picrosirius red staining. Strong, positive correlations were found between the photoacoustic average signal and picrosirius red-stained area at all time points pre- and post-injury. This supports the hypothesis that collagen content within the tendon can be accurately measured via photoacoustic ultrasound in vivo. An earlier study also observed significant correlations between PSR staining and the photoacoustic signal in excised fibrotic kidneys [18].

Interestingly, when separated by time points, significant differences in the collagen photoacoustic average signal were found between uninjured and post-injury groups that were not present in the picrosirius red staining. The likely explanation is differences in collagen alignment throughout injury and healing affected the overall ultrasound and photoacoustic signals. Collagen exhibits anisotropic optical scattering due to its fibrillar structure, meaning light is preferentially scattered in the direction of collagen fiber alignment [21]. This structural change can influence the light distribution within the tissue, and photoacoustic signals are detected. For B-mode imaging, echogenicity is altered by tissue alignment primarily through changes in sound attenuation, propagation, and speed throughout tissue [20]. The moderate to strong correlations found between the collagen photoacoustic signal and echogenicity emphasize that the photoacoustic signal for collagen was likely affected by alignment in this model. While the mechanisms behind alignment affecting echogenicity are slightly different, the result of unaligned tissue inhibiting the overall ultrasound signal remains the same. When examining the whole tendon compared to the injury site alone, the photoacoustic signal exhibited a slightly stronger correlation to PSR staining within the injury site, where the alignment in the tendon changes the most throughout healing. This observed difference could be attributed to compounding factors: (1) most compositional changes during tendon healing occur at the injury site, and (2) the reduction in alignment at the injury site exacerbates the reduction in the collagen signal [3,4,22]. Thus, when utilizing photoacoustic ultrasound for in vivo measures of the tendon, it is important to consider the effects of unaligned collagen fibers that may reduce the overall signal.

Surprisingly, our study only found differences in picrosirius red staining at 1 week post-injury, during the inflammatory phase of healing. Previously in the literature a wide range of reductions in collagen concentration have been described during the inflammatory phase, from 20 to 80% compared to uninjured values [5,6,7]. In the present study, the picrosirius red-stained area at 1 week post-injury decreased by roughly 15% in the whole tendon and 30% when isolating the injury site. However, there were no differences in PSR staining between uninjured tendons and 3 and 6 weeks post-injury, unlike similar studies that showed collagen content plateaued at 90% of uninjured concentrations during tendon healing [8]. These observed differences could be due to the methodologies used to determine collagen content; the cited studies used hydroxyproline to estimate collagen within the tissue, which may be more sensitive to changes [23]. However, hydroxyproline requires tissue digestion for a colorimetric assay, as opposed to imaging sections of tendons stained by picrosirius red. Staining and imaging are more relevant to this study as they allow for regional assessment of collagen content. When considering the strong correlations between the photoacoustic collagen signal and picrosirius red staining, these findings support the validity of photoacoustic ultrasound as a non-invasive tool for estimating collagen content within the tendon. However, further investigation into the effects of collagen alignment on photoacoustic ultrasound may be necessary for more accurate interpretations.

This study is not without limitations. While we did identify effects of collagen alignment on the photoacoustic signal, with a reduction in signal correlating to a reduction in echogenicity, the exact mechanisms of this reduction are still unclear. Due to the nature of a rat Achilles tendon acute rupture model, collagen alignment is closely tied to collagen content within the tissue, with alignment and collagen content significantly decreasing immediately following injury and slowly increasing over the course of healing. One potential solution is to develop phantoms of aligned and unaligned collagen matrices through electrospinning, and varying collagen concentrations and alignment in several phantoms to help titrate the contribution of each to the overall signal [24,25]. Picrosirius red staining was used to estimate collagen content; however, this does not give an exact measure of concentration within the tissue, instead, it primarily identifies if collagen is present or not, giving area % measurements. This study utilized a linear mixing model, assuming the signal of each component is directly proportional to its concentration, which may not be entirely accurate in complex biological tissues where interactions between different components could lead to off-target effects. This approach assumes that signals from different components do not interfere with each other, which might not hold true in cases with overlapping wavelengths [26]. The NIR spectrum was utilized for this study, where overlap can occur due to the smaller range of available wavelengths. A linear mixing model may have difficulties where the light attenuation varies significantly within the tissue, as it does not account for this depth-dependent signal attenuation [27]. However, this can be partly accounted for by increasing TGC at lower depths in the tissue, which was utilized in this study [27]. Future studies can improve this unmixing method by increasing the range of available wavelengths or using a nonlinear mixing model that accounts for the interactions between components.

In conclusion, this study explored a novel application of photoacoustic ultrasound as an in vivo technique for assessing tendon healing. The study offers valuable insights into the potentials and limitations of photoacoustic ultrasound for assessing collagen. The strong correlation between collagen content and photoacoustic signal suggests that this modality can be a non-invasive in vivo tool for assessing collagen content during tendon healing, albeit influenced by collagen fiber alignment. Further studies are warranted to elucidate the impact of collagen fiber alignment on the ultrasound signal. The findings of this study underline the importance of continued investigation in the field of photoacoustic imaging to optimize its applications for monitoring tendon injury, healing, and disease.

## Figures and Tables

**Table 1:** Statistical analysis of the correlation between collagen photoacoustic measurements and picrosirius red area percentage in both tendon and injury areas. The data are presented as correlation coefficients (r), coefficients of determination (r^2^), and significance levels (*p*-values). A strong positive correlation was found between PA average measurements and PSR area percentages in both tendon (r = 0.642, r^2^ = 0.412, *p* < 0.001) and injury areas (r = 0.721, r^2^ = 0.519, *p* < 0.0001).

Comparison	Correlation Coefficient (r)	Coefficient of Determination (r^2^)	Significance (*p*)
Collagen PA Avg vs. PSR Area % (Tendon)	0.642	0.412	0.0002
Collagen PA Avg vs. PSR Area % (Injury)	0.721	0.519	<0.0001

**Table 2:** Correlation between collagen photoacoustic average measurements and echogenicity average measurements in both tendon and injury sites. Correlation coefficients (r), coefficients of determination (r^2^), and *p*-values are presented for each comparison. In the tendon, a strong positive correlation is observed (r = 0.749, r^2^ = 0.551, *p* < 0.0001). Similarly, in the injury sites, a positive correlation is reported (r = 0.603, r^2^ = 0.397, *p* = 0.0003).

Comparison	Correlation Coefficient (r)	Coefficient of Determination (r^2^)	Significance (*p*)
Collagen PA Avg vs. Echo Avg (Tendon)	0.749	0.551	<0.0001
Collagen PA Avg vs. Echo Avg (Injury)	0.603	0.397	0.0003

## Data Availability

Data set will be made available upon request to the corresponding author.

## References

[1] Lin T.W., Cardenas L., Soslowsky L.J. (2004). Biomechanics of tendon injury and repair. J. Biomech..

[2] Kirchgesner T., Larbi A., Omoumi P., Malghem J., Zamali N., Manelfe J., Lecouvet F., Berg B.V., Djebbar S., Dallaudière B. (2014). Drug-induced tendinopathy: From physiology to clinical applications. Jt. Bone Spine.

[3] Sharma P., Maffulli N. (2006). Biology of tendon injury: Healing, modeling and remodeling. J. Musculoskelet. Neuronal Interact..

[4] Sharma P., Maffulli N. (2005). Tendon injury and tendinopathy: Healing and repair. J. Bone Jt. Surg. Am..

[5] Aro A., Perez M., Vieira C., Esquisatto M., Rodrigues R., Gomes L., Pimentel E. (2015). Effect of *Calendula officinalis* cream on achilles tendon healing. Anat. Rec..

[6] Akamatsu F.E., Saleh S.O., Teodoro W.R., da Silva A.Q., Martinez C.A.R., Duarte R.J., de Andrade M.F.C., Jacomo A.L. (2014). Experimental model of Achilles tendon injury in rats. Acta Cir. Bras..

[7] Vieira C.P., De Aro A.A., Guerra F.D.R., De Oliveira L.P., Almeida M.D.S.D., Pimentel E.R. (2013). Inflammatory process induced by carrageenan in adjacent tissue triggers the acute inflammation in deep digital flexor tendon of rats. Anat. Rec..

[8] Goldfarb C.A., Harwood F., Silva M.J., Gelberman R.H., Amiel D., Boyer M.I. (2001). The effect of variations in applied rehabilitation force on collagen concentration and maturation at the intrasynovial flexor tendon repair site. J. Hand Surg..

[9] Fouré A. (2016). New imaging methods for non-invasive assessment of mechanical, structural, and biochemical properties of human achilles tendon: A mini review. Front. Physiol..

[10] Bey M.J., Derwin K.A. (2012). Measurement of in vivo tendon function. J. Shoulder Elb. Surg..

[11] Heinemeier K.M., Kjaer M. (2011). In vivo investigation of tendon responses to mechanical loading. J. Musculoskelet. Neuronal Interact..

[12] Steinberg I., Huland D.M., Vermesh O., Frostig H.E., Tummers W.S., Gambhir S.S. (2019). Photoacoustic clinical imaging. Photoacoustics.

[13] Riggin C.N., Schultz S.M., Sehgal C.M., Soslowsky L.J. (2019). Ultrasound Evaluation of Anti-Vascular Endothelial Growth Factor–Induced Changes in Vascular Response Following Tendon Injury. Ultrasound Med. Biol..

[14] Riggin C.N., Weiss S.N., Rodriguez A.B., Raja H., Chen M., Schultz S.M., Sehgal C.M., Soslowsky L.J. (2022). Increasing Vascular Response to Injury Improves Tendon Early Healing Outcome in Aged Rats. Ann. Biomed. Eng..

[15] Lee H.D., Shin J.G., Hyun H., Yu B.-A., Eom T.J. (2018). Label-free photoacoustic microscopy for in-vivo tendon imaging using a fiber-based pulse laser. Sci. Rep..

[16] Sekar S.K.V., Bargigia I., Mora A.D., Taroni P., Ruggeri A., Tosi A., Pifferi A., Farina A. (2017). Diffuse optical characterization of collagen absorption from 500 to 1700 nm. J. Biomed. Opt..

[17] Wagner A.L., Danko V., Federle A., Klett D., Simon D., Heiss R., Jüngert J., Uder M., Schett G., Neurath M.F. (2021). Precision of handheld multispectral optoacoustic tomography for muscle imaging. Photoacoustics.

[18] Hysi E., He X., Fadhel M.N., Zhang T., Krizova A., Ordon M., Farcas M., Pace K.T., Mintsopoulos V., Lee W.L. (2020). Photoacoustic imaging of kidney fibrosis for assessing pretransplant organ quality. JCI Insight.

[19] Freedman B.R., Sarver J.J., Buckley M.R., Voleti P.B., Soslowsky L.J. (2014). Biomechanical and structural response of healing Achilles tendon to fatigue loading following acute injury. J. Biomech..

[20] Riggin C.N., Sarver J.J., Freedman B.R., Thomas S.J., Soslowsky L.J. (2014). Analysis of collagen organization in mouse achilles tendon using high-frequency ultrasound imaging. J. Biomech. Eng..

[21] Janko M., Davydovskaya P., Bauer M., Zink A., Stark R.W. (2010). Anisotropic Raman scattering in collagen bundles. Opt. Lett..

[22] Freedman B., Gordon J., Castro L. (2014). The Achilles tendon: Fundamental properties and mechanisms governing healing. Muscle Ligaments Tendons J..

[23] Samuel C.S., Becker G.J., Hewitson T.D. (2009). Determination of Collagen Content, Concentration, and Sub-types in Kidney Tissue. Kidney Research: Experimental Protocols.

[24] Riching K.M., Cox B.L., Salick M.R., Pehlke C., Riching A.S., Ponik S.M., Bass B.R., Crone W.C., Jiang Y., Weaver A.M. (2014). 3D collagen alignment limits protrusions to enhance breast cancer cell persistence. Biophys. J..

[25] Law J.X., Liau L.L., Saim A., Yang Y., Idrus R. (2017). Electrospun Collagen Nanofibers and Their Applications in Skin Tissue Engineering. Tissue Eng. Regen. Med..

[26] Keshava N., Mustard J.F. (2002). Spectral unmixing. IEEE Signal Process. Mag..

[27] Grasso V., Holthof J., Jose J. (2020). An Automatic Unmixing Approach to Detect Tissue Chromophores from Multispectral Photoacoustic Imaging. Sensors.

